# Recent developments of nano-structured materials as the catalysts for oxygen reduction reaction

**DOI:** 10.1186/s40580-018-0144-3

**Published:** 2018-04-30

**Authors:** SungYeon Kang, HuiJung Kim, Yong-Ho Chung

**Affiliations:** Department of Chemical Engineering, Hoseo University, Hoseoro79bungil20, Baebang, Asan, Chungnam 336-795 Republic of Korea

**Keywords:** Electrocatalyst, Nano-structured material, Oxygen reduction reaction, Pt alloys, Non-Pt catalyst

## Abstract

Developments of high efficient materials for electrocatalyst are significant topics of numerous researches since a few decades. Recent global interests related with energy conversion and storage lead to the expansion of efforts to find cost-effective catalysts that can substitute conventional catalytic materials. Especially, in the field of fuel cell, novel materials for oxygen reduction reaction (ORR) have been noticed to overcome disadvantages of conventional platinum-based catalysts. Various approaching methods have been attempted to achieve low cost and high electrochemical activity comparable with Pt-based catalysts, including reducing Pt consumption by the formation of hybrid materials, Pt-based alloys, and not-Pt metal or carbon based materials. To enhance catalytic performance and stability, numerous methods such as structural modifications and complex formations with other functional materials are proposed, and they are basically based on well-defined and well-ordered catalytic active sites by exquisite control at nanoscale. In this review, we highlight the development of nano-structured catalytic materials for ORR based on recent findings, and discuss about an outlook for the direction of future researches.

## Background

According to the change of global demands to green energy as the substitute of fossil fuel, electrocatalysts with high efficiency have been received great attentions to various researchers in the field of renewable energy [1–3]. Especially, oxygen reduction reaction (ORR) was crucial issue in polymer electrolyte membrane (PEM) fuel cell, because slow kinetics of cathodic reaction related with oxygen reduction limits overall system efficiency. PEM fuel cell has been regarded as the most promising energy system for the environmental friendly vehicles, resulted from high energy density and fast refueling. As a result, several commercial PEM fuel cell vehicles were launched by global automobile makers, but development of electrocatalyst with high efficiency and low cost was remained as the ongoing task for the market expansion. Platinum (Pt) and Pt-based materials were mainly utilized in the fuel cell as the catalysts due to the fact that they were considered as the most effective electrocatalysts for ORR [4, 5], but high cost of Pt and low durability of support materials such as carbon particle prevent the practical commercialization [6]. The platinum contents of commercial Pt/C catalyst were ranged from 10 to 60 wt%, and loading amount of Pt was about 0.4 ~ 0.8 mg_pt_/cm^2^. The set target of the Department of Energy (DOE) is 0.125 mg_pt_/cm^2^ for the practical commercialization until 2020, and researches for reducing Pt consumption have been attempted. Also, there was another problem in addition to high cost and catalytic performance, and it was long-term stability. Pt-based catalysts have the problems related with Pt dissolution and corrosion of carbon support due to the acidic operating condition. Therefore, development of cost-effective electrocatalyst for fuel cell was governed by maximizing mass and specific activities, and minimizing Pt usage with high stability.

Various researches have been attempted to improve those problems above mentioned. Common research directions were reduction of Pt consumption in electrochemical cell, even though there were a lot of approaching methods to achieve high cost-efficiency and durability. Increasing surface area by reducing the size of Pt particle was the most simple and effective method to minimize Pt consumption, because catalytic reaction is operated in the surface of catalyst, however size controlling to the scale of a few nanometers has the practical limitation caused by the severe aggregation due to the high surface energy of nanoparticles. To synthesize Pt nanoparticle without aggregation, site-specific synthetic methods in the template were attempted in several research groups [7, 8]. Graphene as the template for the Pt nanoparticle has the planar structure with sp2-bonded carbon atoms, and it has excellent conductivity and stability, resulted in the widely applications to energy conversion system including fuel cell.

Electrodes of fuel cell were usually formed with complex composed of catalysts and supporting materials for preventing aggregation and maximizing surface area, and carbon black has been utilized as supporting materials due to its high conductivity and porosity. But, this materials could be easily corroded in the acidic working condition, resulted in fast degradation of cell performance. So, there have been various efforts to develop alternating materials with high durability for fuel cell, such as TiO_2_, SiC, and CeO_2_ [9–11]. Another method to reduce Pt consumption was to enhance the performance of electrocatalyst, and results by the formation of alloy using Pt and other metals showed different activity according to the facets and bonding structure [12–14]. For example, octahedral Pt_x_Ni_1−x_ alloy showed excellent performance than conventional Pt/C catalysts alloy that Ni atoms were segregated in (111) facets [15].

In addition, numerous approaching methods based on Pt-free catalyst have been attempted to develop cost-effective materials [16–19]. Though several materials showed possibilities to applications as the catalysts in terms of catalytic performance, serious problems such as catalytic activity and durability should be improved for practical commercialization [19].

In this review, we investigate and discuss about the electrocatalysts with high cost-efficiency and durability for fuel cell based on recent findings, and highlight important results. Also, we will conclude this review by arranging current challenging and future perspective for the catalysts of fuel cell.

## Downsizing Pt nanoparticle

### Pt/graphene oxide (GO) nanocomplex

Among the methods for increasing efficiency of platinum catalysts, the method to increase surface area was the most efficient way. It has been shown that reducing the size of the particles to the nanometer greatly enhanced the performance of the catalysts. However, effective supporting materials were needed because increasing the surface area of platinum particles will have an aggregation effect between platinum molecules [20–23].

**Fig. 1:**
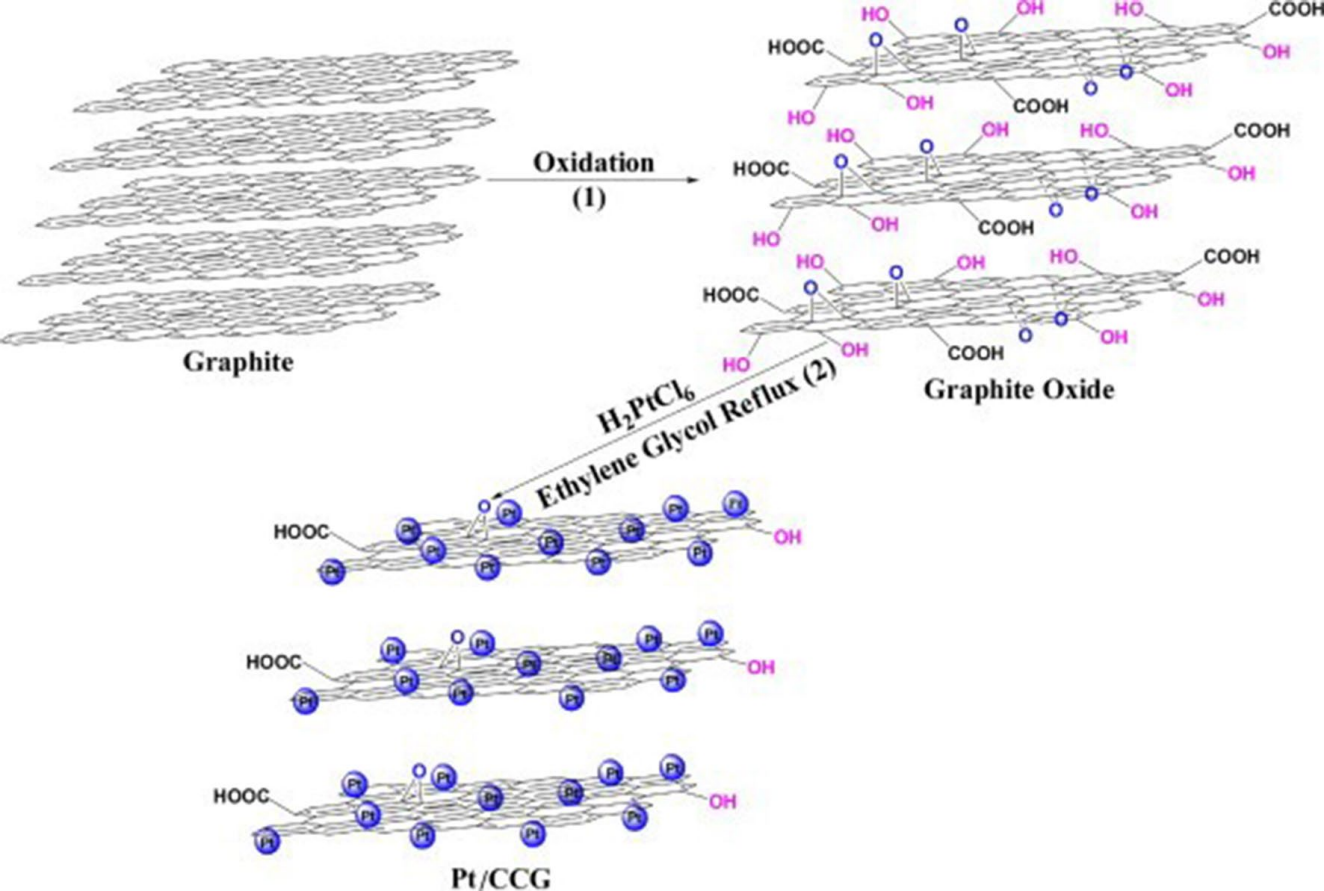
Fabrication process of Pt/GO hybrid sheet, (1) oxidation of graphite powder to GO, (2) formation of Pt/GO hybrid sheet (reprinted with permission from Ref. [32] Copyright @ 2010 Elsevier)

**Fig. 2:**
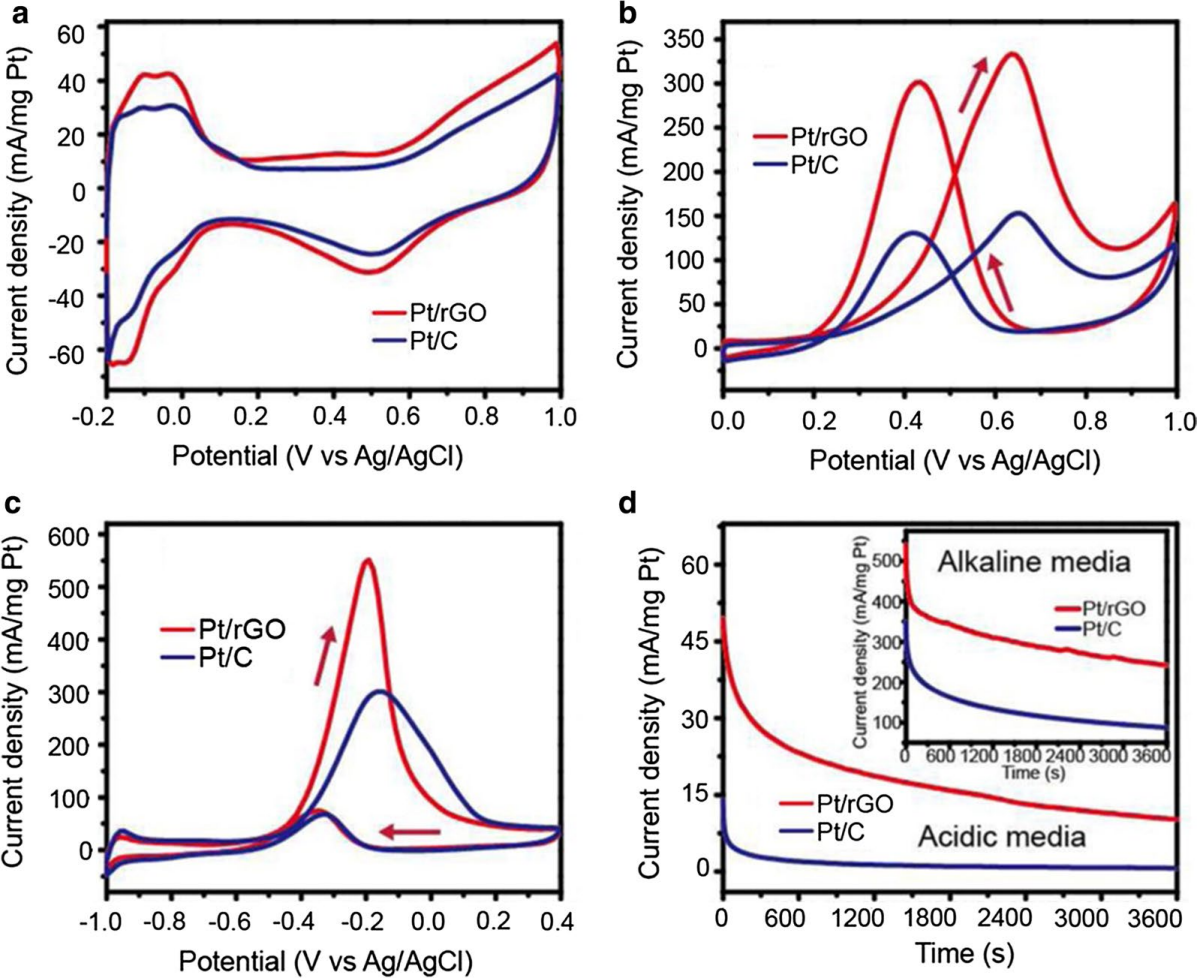
CV curve of fabricated Pt/rGO and conventional Pt/C in **a** 0.5 M N_2_-saturated H_2_SO_4_, **b** 0.5 M H_2_SO_4_ + 0.5 M CH_3_OH, and **c** 0.5 M KOH + 0.5 M CH_3_OH. **d** 0.5 M H_2_SO_4_ + 0.5 M CH_3_OH (reprinted with permission from Ref. [30] Copyright @ 2015 American Chemical Society)

Graphene is a 0.34 nm single layer structure and is suitable as a supporting material of platinum because of its properties such as excellent thermal, electrical conductivity, high electron transfer rate, and excellent chemical resistance [24–29]. Graphene effectively prevents the aggregation of platinum by allowing it to be placed between pores in the increased surface area for the efficiency of the catalyst. Also, graphene is used as a supporting material because it is active in acidic and alkaline solutions better than carbon black and also has long-term stability [30, 31]. Figure 1 showed representative synthetic process of Pt/GO hybrid sheet. Pre-treated GO sheets have numerous functional groups on the surfaces such as –O, –OH, and –COOH, and they could provide stable site for the synthesis of Pt nanoparticles. Nano-sized Pt particle could be located on the graphene sheet with well-dispersed shape, and that complex showed enhanced catalytic performance [32], compared with Pt/multi-wall carbon nanotube that was considered as an excellent promising catalytic materials for fuel cell [33, 34]. Wu et al. reported another method for the synthesis of Pt–GO hybrid materials based on substitution of sacrificial materials [30]. MnO_x_/GO nanocomplex produced by laser ablation in liquids were reacted with PtCl_6_^2−^, and Pt/GO complex could be easily synthesized by the substitution of MnO_x_ to Pt atoms. As a result, they demonstrated a better performance based on fabricated nanocomplex than conventional Pt/C electrocatalyst as shown in Fig. 2. Also, several researches based on graphene such as GO modified Pt nanoflower [35], Pt nanoparticle on defective graphene [36], and sandwich-structured graphene/Pt/graphene [7] have been researched to reduce Pt consumption.

### Pt/GO/CB nanocomplex

**Fig. 3:**
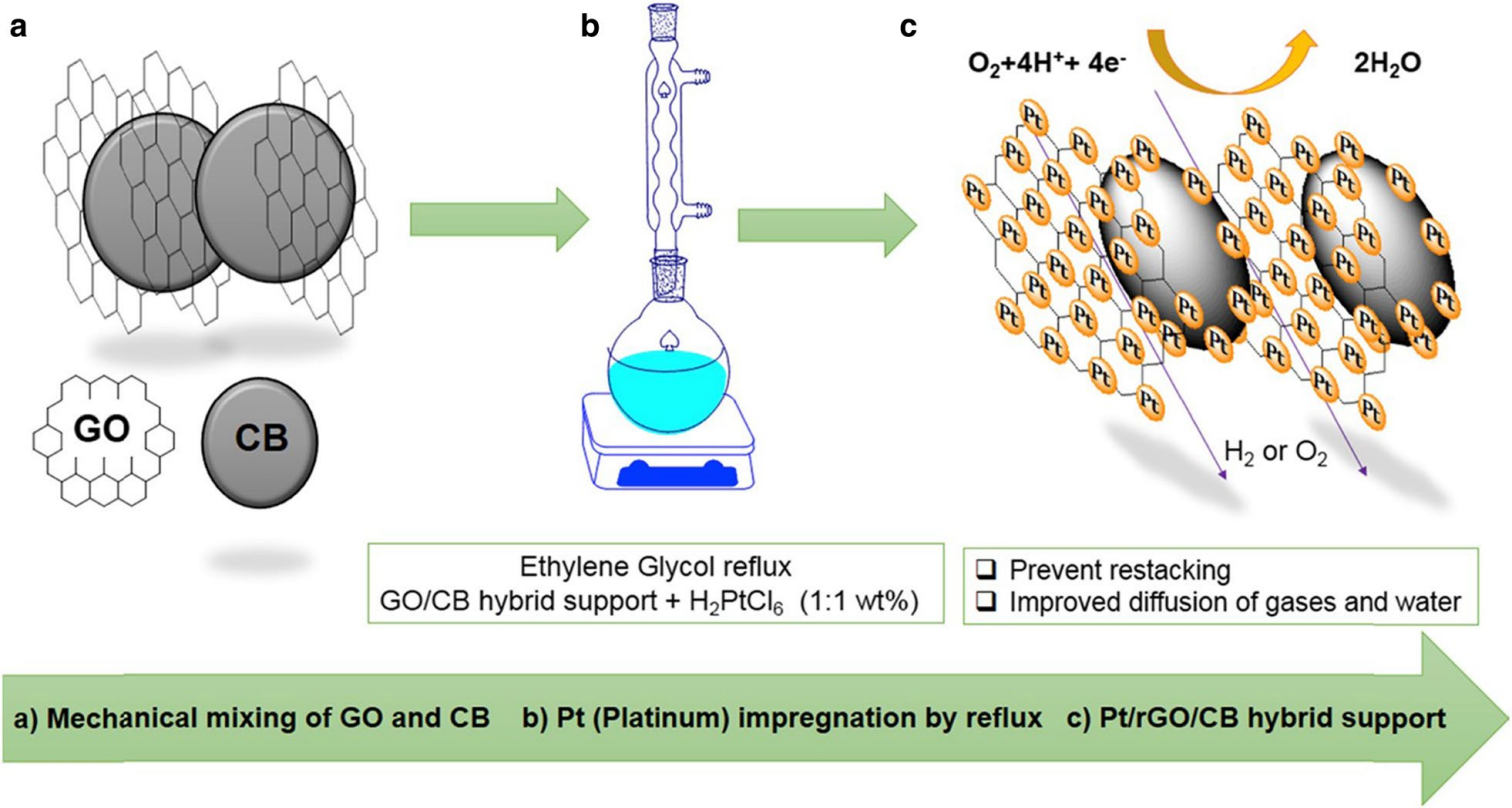
Fabrication process and structure of Pt/GO/CB hybrid support (reprinted with permission from Ref. [39] Copyright @ 2017 Elsevier)

During fabrication process of Pt/GO nanocomplex, the graphene sheet combined with Pt nanoparticle has a tendency to be layered horizontally, resulted in the formation of stacked catalyst layer. This tendency is a fatal disadvantage to the performance of the electrode including reducing active area of Pt and high diffusion resistance to the reactants in operation process [37, 38]. Carbon black (CB) as a supporting materials can be an alternative to relieve stacking effects due to the high electrical conductivity and increase of a surface area (Fig. 3). Spherical CB is added between layer of graphene sheets to prevent stacking effects and the electrical activity of CB helps the final catalytic performance. The effects of graphene and carbon black result in reducing platinum usage, since they can improve the performance of the catalyst and power output, and provide high utilization of platinum. The highest current produced when the catalyst was synthesized at a ratio of GO and CB with a rate of 75:25, and comparing the efficiency of platinum in H00 (Pt/CB), H100 (Pt/GO), and H75 (Pt/GO/CB) showed that H75 has the highest efficiency as the 2.58 KW/g Pt. When the catalyst is synthesized at a ratio of H75, the amount of platinum consumption could be effectively reduced [39].

### Pt/GO/TiO_2_ nanocomplex

**Fig. 4:**
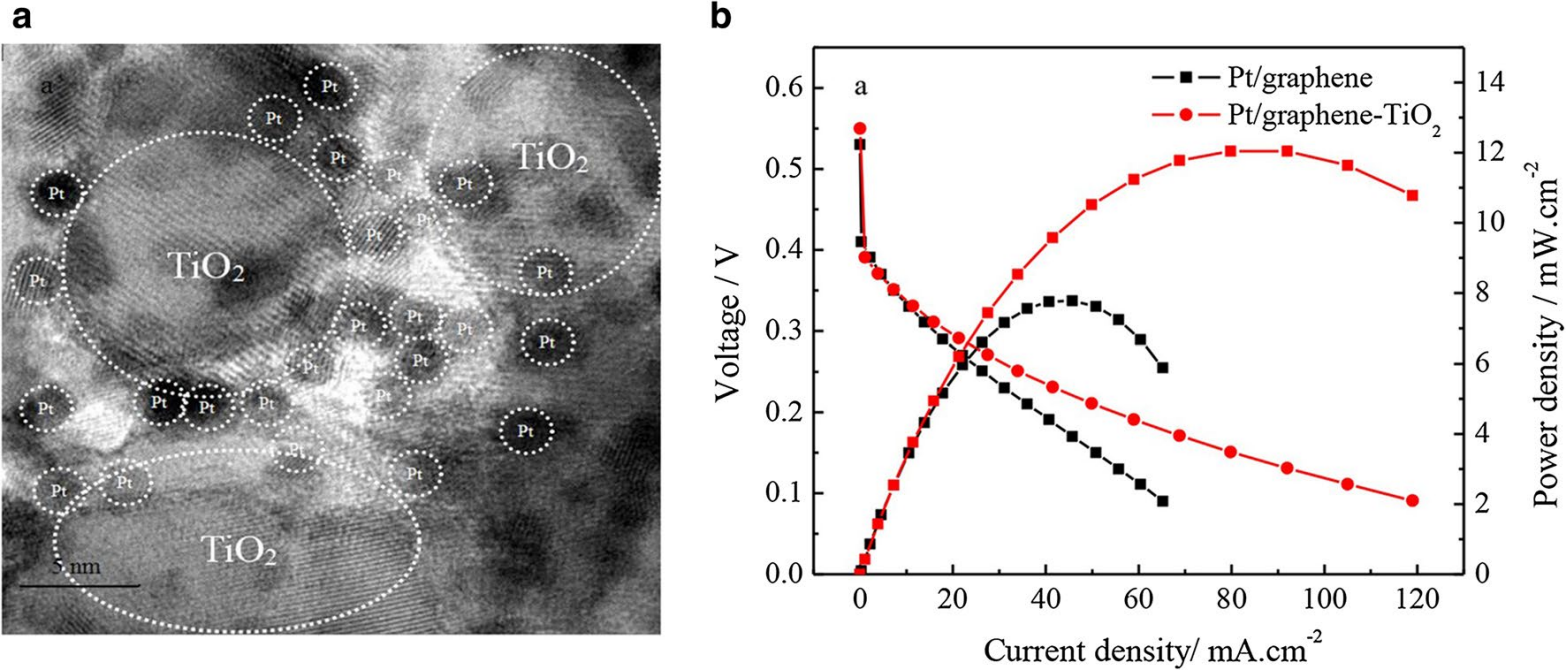
**a** TEM image of Pt/graphene/TiO_2_ catalyst, **b** single DMFC performance of Pt/graphene and Pt/graphene/TiO_2_ catalyst (reprinted with permission from Ref. [44] Copyright @ 2015 Elsevier)

In case of CB support, it is difficult to secure long-term durability as a catalyst for fuel cells due to the corrosivity of CB in acidic working condition. As described above, it is necessary to add supporting materials between graphene sheets for mitigating the stacking effect. Various alternating materials have been researched to overcome the limitation of CB [9–11, 40, 41]. Especially, metal oxides such as titanium oxide may become substitutes for carbon black due to low price and inherent characteristics related with high stability in working condition of fuel cell [42, 43]. Though the electrical activity of the titanium oxide is lower than the carbon black, it is similar to the power output of the conventional catalysts because of the electrical activity with graphene. The result using titanium oxide showed stability enhancement of 15% (Fig. 4) in comparison with Pt/rGO catalyst and the increase of its power output by 55%. This could be caused by strong interaction between metal and supporting materials, and synergetic effect between TiO_2_ and graphene [44].

### Pt/carbon nanotube nanocomplex

**Fig. 5:**
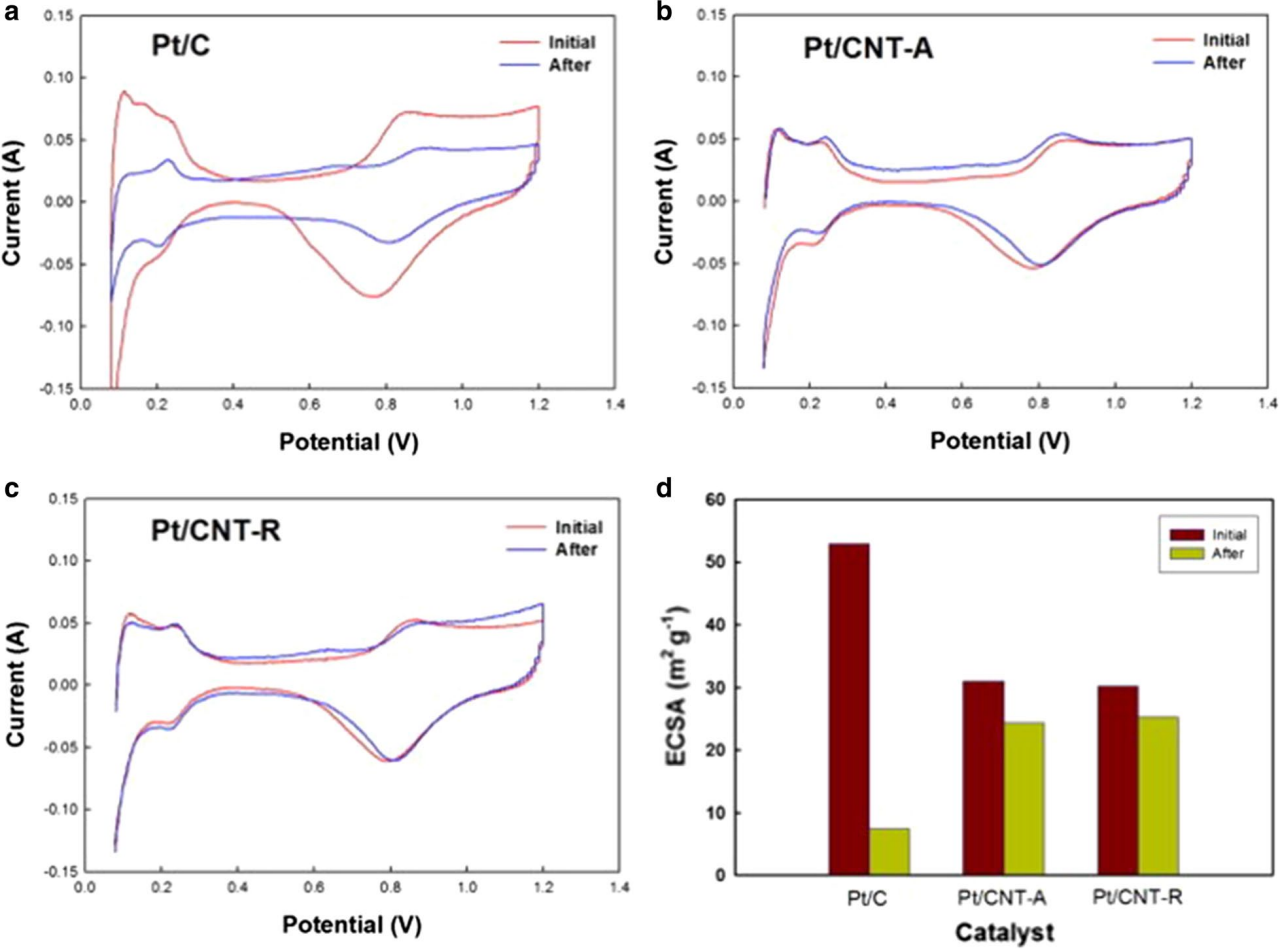
CV measurements of MEAs before and after acceleration test **a** Pt/C, **b** Pt/CNT-A, **c** Pt/CNT-R, **d** comparison of capacitances (reprinted with permission from Ref. [48] Copyright @ 2012 Elsevier)

Another way to replace the carbon black and reduction of Pt consumption is to apply carbon nanotubes (CNT) to the catalyst formation. CNT has the properties of excellent corrosion resistance, electronic conductivity and specific surface area [45, 46], and can prevent the aggregation of platinum, resulting in enhancement of catalytic activity [4, 47]. Also, long-term durability mentioned by the demerit of CB was crucial point to overcome for the commercialization of fuel cell. Lee et al. have reported Pt/CNT catalyst with improved durability for PEM fuel cell [48]. As shown in Fig. 5a, the high initial current was monitored in Pt/C commercial catalyst, but the total current was decreased after acceleration test, and that meant low durability of commercial Pt/C electro-catalyst. However, initial current in case of Pt/CNT catalyst was changed a little after test in Fig. 5b–d. Activity loss about 62.5% was monitored in Pt/C, but only 6.2% variation was occurred in Pt/CNT catalyst. Also, polarization resistance of Pt/CNT was increased only 3%, in contrast with 700% of Pt/C catalyst. Li et al. have reported size-differences between Pt nanoparticles fabricated on GO and multi-wall CNT [32]. Average Pt sizes of Pt/GO and Pt/MWCNT were 2.75 and 3.5 nm, respectively, and it meant that GO was more suitable template material to reduce the size of Pt nanoparticle, in spite of several advantages of CNT.

## Pt-based alloys

**Fig. 6:**
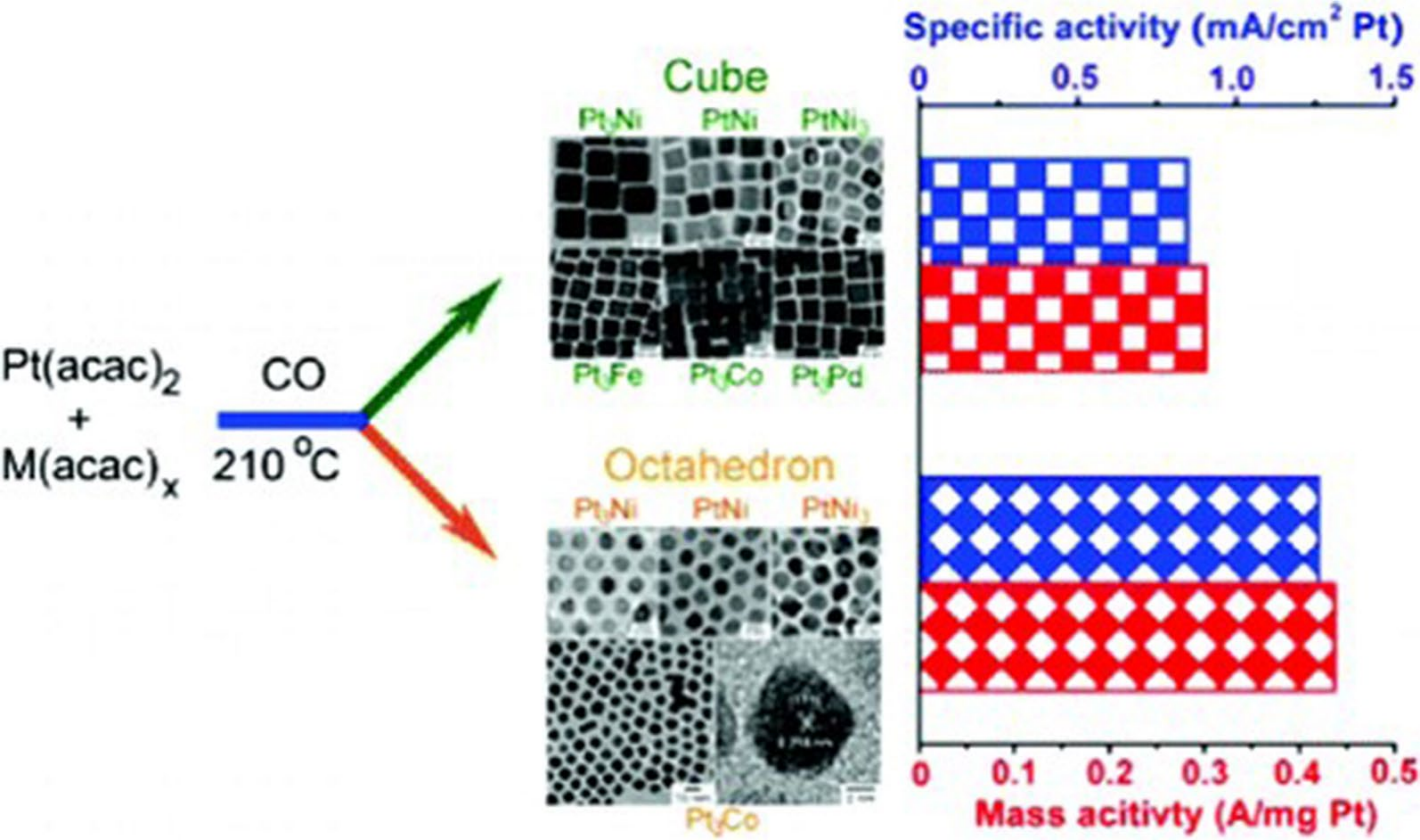
Schematic description of shape controlled synthesizing process. Pt-metal alloys were fabricated by gas reducing agent in liquid solution (GRAILS) method (reprinted with permission from Ref. [52] Copyright @ 2011 American Chemical Society)

Pt-alloys with late transition metals have been firstly researched from the early 1980s, and various results reported that alloys with Ni and Co have the higher activities than Pt-based catalyst in terms of ORR [49], but low stabilities of those researches related with the dissolution during operation process were ongoing research topics. Recently, numerous attempts to synthesize Pt-alloys using rare earth and early transition metals have been operated by the theoretical simulation and specific experimental results. In 2009, Greeley et al. proposed calculated data named volcano plot for predicting activities of Pt-based alloy materials [50], and this plot showed reliable correlation with recent reported findings [51]. Reported researches have showed meaningful cost-effective performances, but suitable methods for practical commercialization such as simple mass production were still remained ongoing researches. Wu et al. reported effective synthetic method for Pt-metal (Co, Fe, Ni, Pd) alloys [52]. Four kinds of Pt–M alloys were synthesized with same process, and shapes were controlled as cubic and octahedral structure to form well-defined morphologies because they usually play key roles in catalytic reactions (Fig. 6). Also, the ORR mass activities of fabricated octahedral Pt_3_Ni catalyst achieved 0.44 A/mg_pt_, and it was higher result than conventional Pt/C catalysts.

### Pt–Ni alloys

**Fig. 7:**
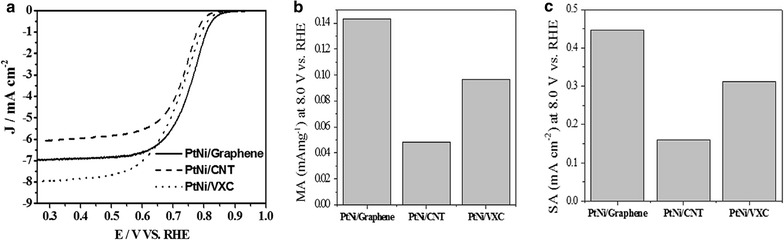
**a** ORR activities of Pt–Ni/VXC, Pt–Ni/CNT, and Pt–Ni/graphene catalysts in O_2_ saturated 0.5 M H_2_SO_4_ with a scan rate of 5 mV/s at room temperature, **b** mass and **c** specific activities of three catalysts at 0.8 V in a 0.5 M H_2_SO_4_ electrolyte (reprinted with permission from Ref. [58] Copyright @ 2016 Elsevier)

Intermediates species such as O, OH, OOH molecules could be easily adsorbed into the platinum surface in the operation process of the oxygen reduction reaction (ORR), and it leads to decrease total active sites for oxygen adsorption, resulted in decline of ORR activities. Platinum and oxygen molecules have mixed potential through the formation of oxide, and the formed oxide prevents ORR [53, 54]. Oxygen molecules in the surface may be adsorbed by secondary transition metals such as Fe, Cu, and Ni in the alloy with platinum [55]. Platinum has high binding energy between oxygen molecules and the surface. The D-band center of the platinum surface has a higher Fermi level than the alloy catalyst. When the alloys are combined, electron transfer takes place from the metal to platinum, resulting in a larger D-band and lower Fermi level [56, 57]. Also, when those alloys were combined with carbon support such as carbon black, graphene and carbon nanotubes, catalyst performance could be enhanced by synergetic effects. Figure 7 indicated ORR activities according to the change of supporting materials as Vulcan XC-72 (CB), graphene, and CNT. Among three results, the catalysts using graphene support showed the highest value, but its stability was lower than other two cases [58].

Chen et al. developed Pt_3_–Ni bimetallic nanocrystals for electrocatalyst [59], and it showed 5.7 A/mg_pt_ in mass activity. This nanostructure has the special open-framework with crystalline Pt–Ni alloy (111), resulting in high stability, more active than commercial Pt/C catalysts, even after 10,000 rounds of voltage cycles. Also, in 2015, Huang et al. reported novel Pt–Ni octahedral alloy catalyst supported by carbon with transition metals [60]. Overall structure were represented as M–Pt_3_Ni/C, where M is applied with vanadium, chromium, manganese, iron, cobalt, molybdenum tungsten, or rhenium. Among them, Mo–Pt_3_Ni/C recorded the best ORR performances which were a specific activity of 10.3 mA/cm^2^ and a mass activity of 6.98 A/mg_Pt_, respectively, in contrast of the values of commercial Pt/C catalysts (0.127 mA/cm^2^ and 0.096 A/mg_Pt_).

### Pt–Cu alloys

**Fig. 8:**
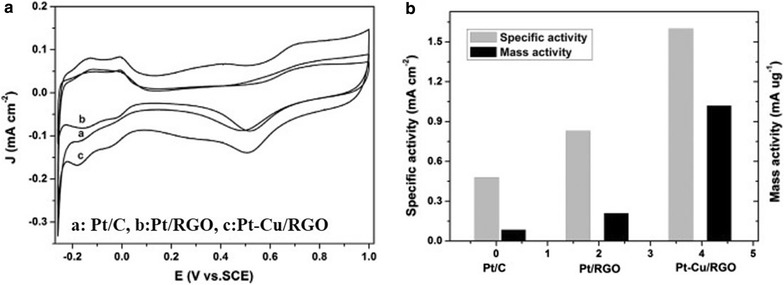
**a** Cyclic voltammograms of Pt/C, Pt/RGO, and Pt–Cu/RGO catalysts in 0.5 M H_2_SO_4_ at a scan rate of 50 mV/s. **b** Specific and mass activities of the Pt/C, Pt. RGO, and Pt–Cu/RGO catalysts modified GC electrode at 0.62 V (reprinted with permission from Ref. [67] Copyright @ 2013 Elsevier)

Platinum and copper alloy were also used as catalysts for fuel cell because they have benefits to generate high current in comparison with Pt/C catalysts. Because copper was highly active in the oxidation of the intermediate carbon, Pt–Cu alloy has been considered as promising candidate for the cost-effective catalysts [61]. Various methods based on hydrothermal process have been reported to fabricate Pt–Cu alloys [62, 63], and those methods usually required additional process to make porous Pt-rich structure by removing pre-combined Cu atoms. Yang et al. reported fabrication method based on simple electrodeposition method [64]. They have developed hierarchical Pt–Cu/reduced GO catalyst by one-step fabrication process, in which high electrocatalytic activity of 2.3 times (vs. commercial Pt/C catalyst) and robust durability was achieved. Normally, morphological control using supporting materials with large surface area have been used to enlarge active sites of catalysts. Graphene oxide has not only excellent properties as the supporting materials, but also its numerous functional group in the surface can provide binding site with catalyst nanoparticle. However, natural tendency of graphene to stack horizontally resulted in the decrease of catalytic sites as described above. Fabrication of hierarchical structure using GO could be desirable method to enhance catalytic activities by facilitating transfer ratio and increasing accessibility [65, 66], in addition to other methods based on supporting materials such as carbon black or metal oxide [39, 44]. Results about electrochemical activities according to the species of catalysts complex in Fig. 8a indicated the effects of alloying metal and graphene support [67]. Strong responses corresponding to adsorption and desorption of hydrogen were monitored in all CV curves, but Pt–Cu/RGO catalysts showed the highest value in all cases. Also, normalized values based on Pt loading (mass activities) and electrochemical active surface areas (specific activities) were represented in Fig. 8b, in which Pt–Cu/RGO catalysts showed remarkable catalytic activity compared to Pt/RGO and Pt/C catalysts.

### Catalysts based on Pt-based alloys

**Fig. 9:**
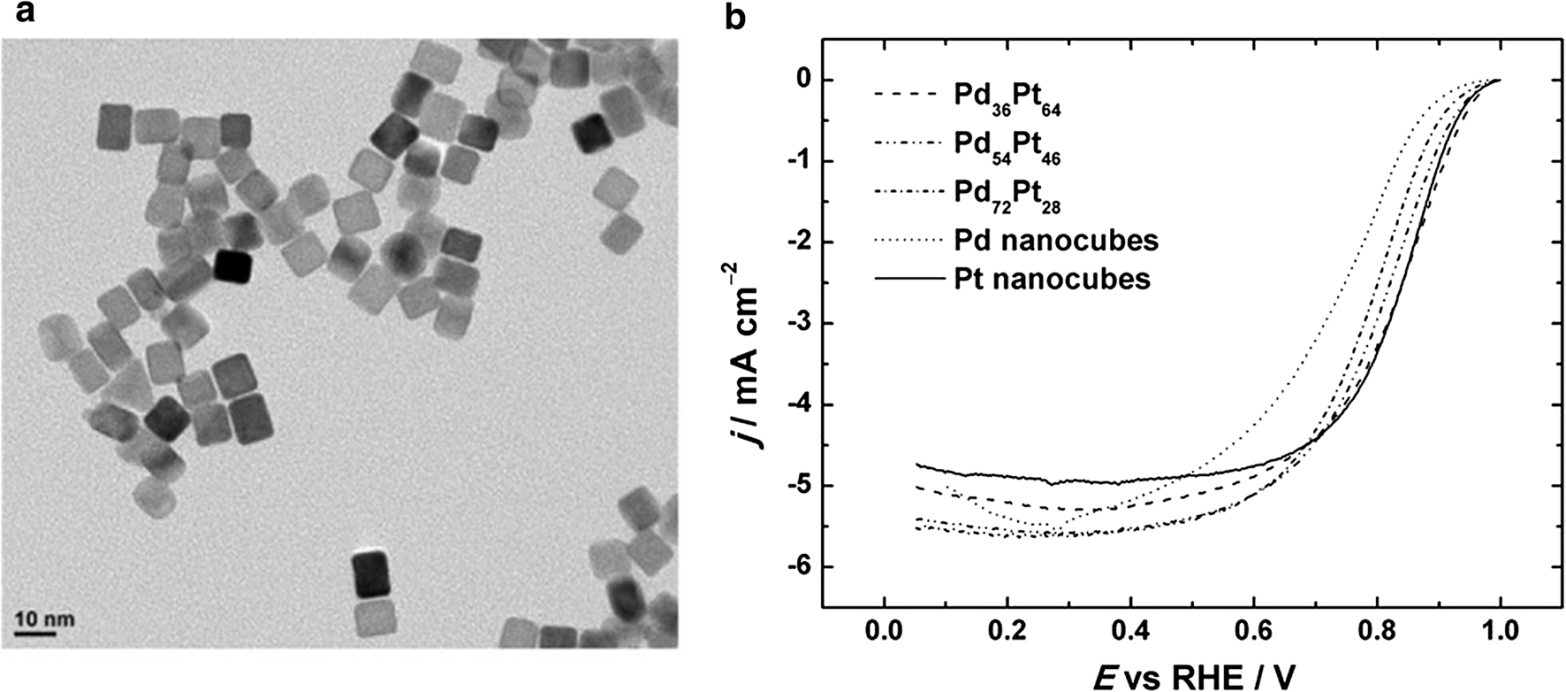
**a** TEM image of Pd–Pt nanocube catalyst, **b** comparison of the RDE results for the ORR (ν = 10 mV/s, ω = 1900 rpm) (reprinted with permission from Ref. [72] Copyright @ 2015 Elsevier)

Besides Pt alloys with Ni and Cu as described above, several researches based on other metals or structural changes have been attempted to achieve low cost and high durability [68, 69]. Zhao et al. reported octahedral core–shell nanocrystals composed of Pd and Pt–Ni, and fabricated nanocrystal achieved 5 times higher mass activities than commercial Pt/C catalysts [70]. Also, another research group developed core–shell tetrapod structures composed of Pd and Pt [71]. This nanostructure with dendritic morphology showed remarkable activity and durability as the 13% loss of initial electrochemical surface area in comparison with the 78% loss that of Pt/C catalyst. In addition to those researches, various researches based on structural control with several non-Pt materials have been attempted including Pd–Pt nanostructure with the shape of cube as shown in Fig. 9 [72], ternary Pt–Pd–Cu sphere [73], dendritic Pt–Ni–P alloy [74], multimetallic nancube [75], dealloyed Pt–Co_x_ [76], core–shell Ni/Fe–Pt [77], octahedral Pt_2_–Cu–Ni [78], and Pt-coated Co nanowire [79]. Even though remarkable scientific results in terms of high activity and stability have been reported from various research groups, the ultimate research direction for fuel cell catalyst should be non-Pt based catalysts, because those results above mentioned were based on the catalytic performance of platinum with high cost.

## Non-Pt catalysts

The penetration of ethanol through polymeric membranes (alcohol crossover phenomenon) could interfere with ORR rate. Also, the combination of oxygen atoms to catalysts is so strong that very slow ORR occurs at the cathode. Pt-based catalysts have been mainly utilized in the practical fuel cells to solve these problems. However, numerous researches to apply non-Pt catalytic materials have been conducted at the same time to overcome disadvantages of Pt-based catalysts such as high price and low stability [80–85].

### Pd-based catalysts

Among the different types of non-platinum catalyst, palladium nanoparticles have high resistance to crossover and excellent ORR performance. The face-centered cubic (fcc) of the palladium has the same atomic size and electrical properties with Pt. Furthermore, the price of Pd is very low than Pt because of abundant deposit [86, 87]. However, Pd has low stability in acidic condition and high temperature than Pt, it should be synthesized with various transition metals and supports to secure stabilities [88, 89]. Especially, Pd–Ni/C catalyst has been considered promising candidate due to the low price, non-toxic, and high ORR response [90].

**Fig. 10:**
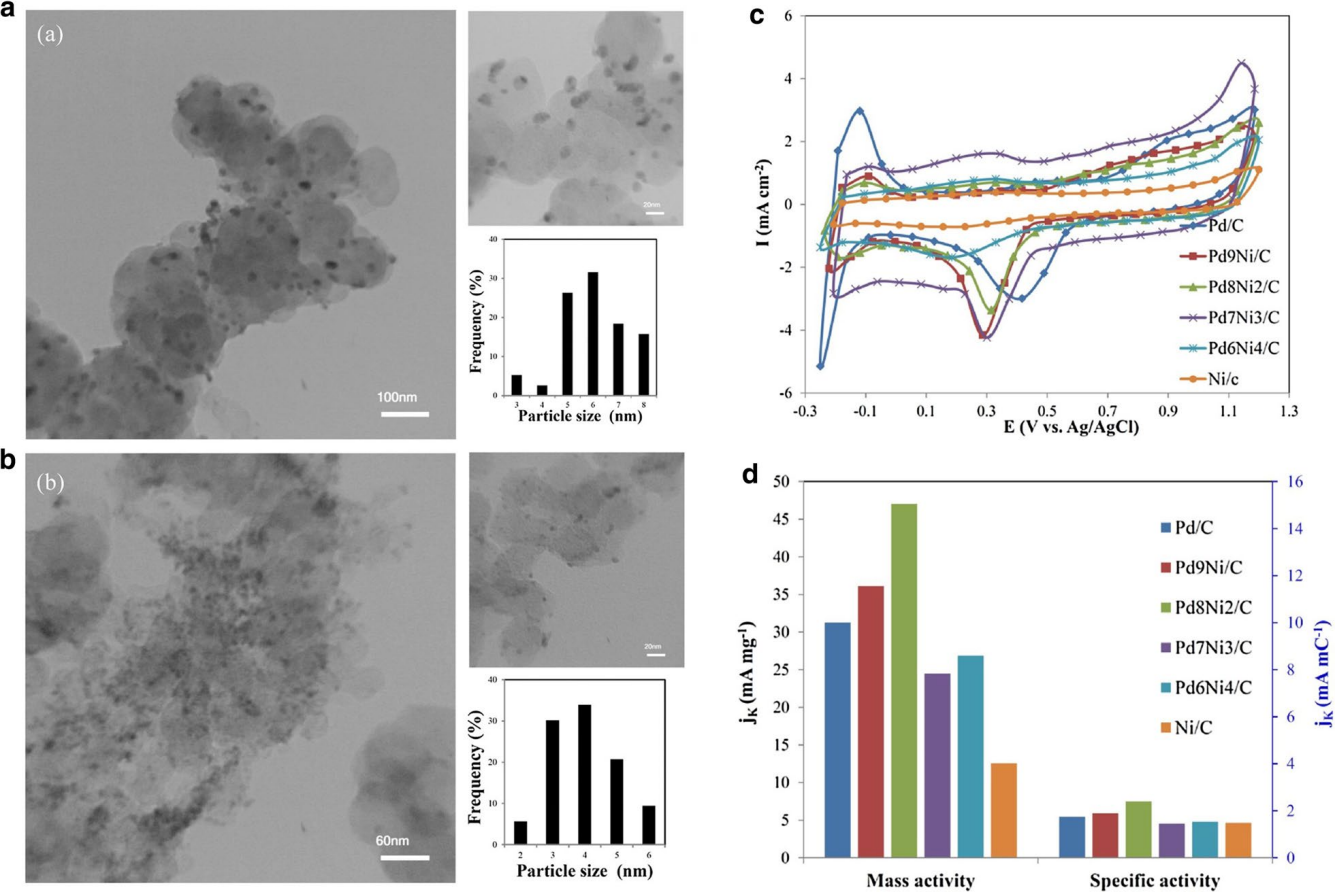
**a** TEM images of Pd/C, **b** Pd–Ni/C, **c** CV curves of the Pd/C, Ni/C, and Pd–Ni/C catalysts according to the atomic ratio in 0.5 M H_2_SO_4_, **d** mass and specific activities of fabricated various electro-catalysts at 0.1 V (reprinted with permission from Ref. [91] Copyright @ 2018 Elsevier)

Recently, Sanij et al. reported fabrication of Pd–Ni/C with superior catalytic performance [91]. Figure 10a, b indicated size differences of Pt/C and Pt_8_Ni_2_/C nanoparticles, and the latter has relatively smaller than the former as 3.6 and 5.5 nm, respectively. Electrochemical activities according to the atomic ratio between Pd and Ni were shown in Fig. 10c, d, in which Pd_8_–Ni_2_/C has the highest activity in all catalysts in addition that Pd–Ni/C catalysts were basically higher than Pd/C. Also, hydrogen adsorption/desorption peaks were observed between − 0.2 and 0.02. As a result, the separation of hydrogen could be restricted by adding Ni to Pd atoms. Those phenomena related with performance improvement of mass and specific activities were caused by synergetic effects between Ni and Pd, nanoscale particle size, and carbon support materials [92, 93]. As a result, Pd–Ni/C has showed high possibilities than other catalysts in terms of activity, resistance to methanol, ORR efficiency, and stability [91].

**Fig. 11:**
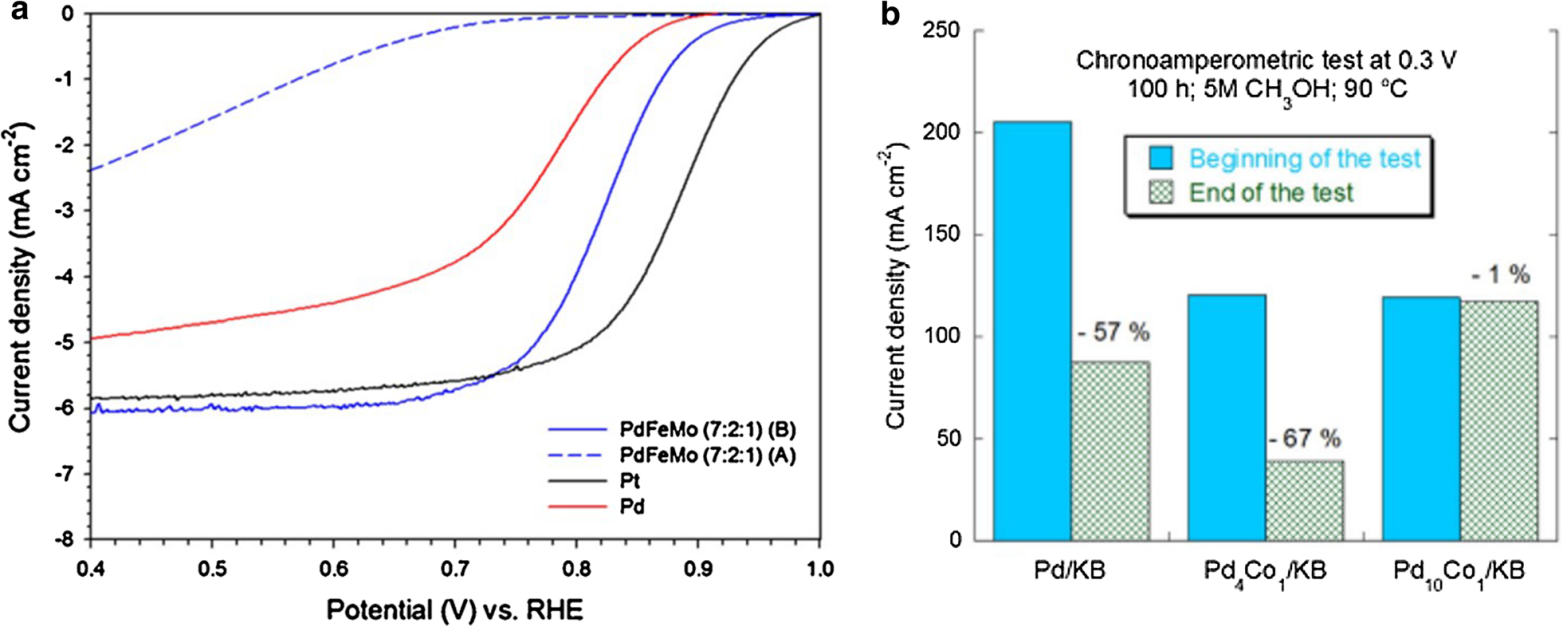
**a** Oxygen reduction reaction (ORR) current densities of Pt, Pd, and Pd–Fe–Mo catalysts. **b** Current density of Pd/C, Pd–Co/C at 0.3 V in chronoamperometry measurements at BOT and EOT (reprinted with permission from Ref. [96, 97] Copyright @ 2018, 2016 Elsevier)

Also, several Pd-based alloy materials with transition metal such as Co and Fe have been researched in various research groups. Tang et al. have developed Pd–Fe/C electrocatalyst and reported the effect of Fe state [94], and Zhang et al. reported results related with the effect of heat treatment to control Pd–Co nanoparticle size which could affect ORR activity [95]. Co atoms combined with Pd could aid to enhance corrosion resistance and durability of the catalyst [96]. Figure 11a showed high stability of Pd–Co/C electrocatalyst, in contrast with considerable differences between BOT and EOT of Pd/C. Compared to the stability of the Pd catalyst, the Pd–Co catalyst was much more stable than that of the Pd. Also, the Pd catalyst has a good property than the commercial catalyst in transmitting methanol, it is more advantageous in terms of cost reduction and increase of energy density as an alternation materials of Pt-based catalysts. Pd–Fe–Mo/C synthesized with the ratio of 7.5:1.5:1.0 at 500 °C showed high activity and durability compared with commercial Pt-based and non-Pt catalysts as shown in Fig. 11b [97].

### Various catalysts based on non-Pt materials

**Fig. 12:**
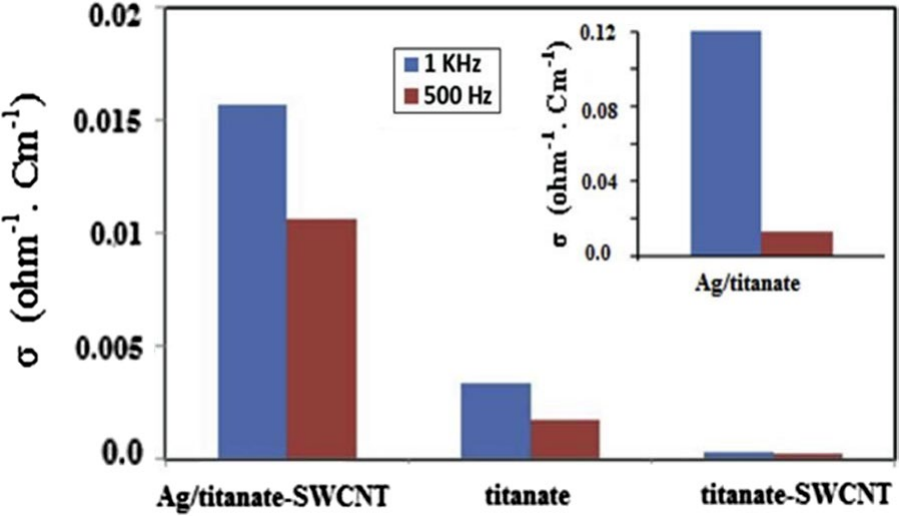
The change in conductivity values of Ag/titanate/SWCNT, titanate, and titanate/SWCNT at 500 Hz, and 1 kHz (reprinted with permission from Ref. [104] Copyright @ 2016 Elsevier)

Besides Pd-based catalysts above mentioned, numerous researches using non-Pt materials have been developed to achieve efficient novel electro-catalysts with low cost [17, 19, 98–100]. Titanate has been investigated to apply catalyst for fuel cell because of its low price and stability. However, it has low conductivity and poor performance compared with commercial catalyst, so it should be made into catalysts with other materials [101, 102]. Carbon nanostructure with larger surface area are often used for catalyst synthesis due to their electrical and structural characteristics [103]. As a result of complex formation, the property of titanate composite as the catalyst could be reinforced in terms of surface area, structural stability, and lifespan. Mohamed et al. reported catalytic performance of Ag/titanate and Ag/titanate-SWCNT [104]. As a non-platinum catalyst, the titanate compound showed high stability, current generation and corrosion resistance. It was due to the fact that the catalytic function of Ag could be improved by the interaction between Ag and titanate and the Ag particle size was reduced by synthesis process with titanate. Also, the electrode catalyst performance was enhanced by uniform distribution (Fig. 12).

**Fig. 13:**
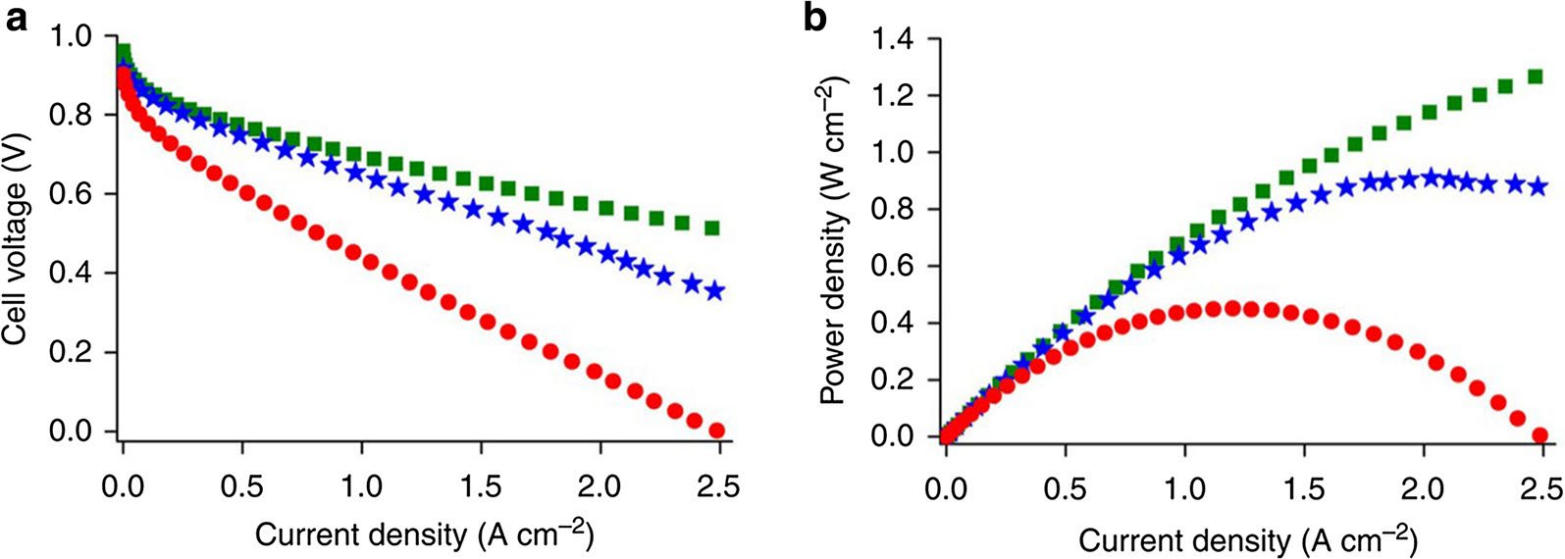
Polarization and power density curves of MEAs using NRE 211 membranes (Fe/Phen/Z8: blue, previous most active Fe-based catalyst: red, commercial Pt-based catalyst: green). **a** Polarization curves for MEAs, **b** power density curves corresponding to polarization curves in **a** (reprinted with permission from Ref. [19] Copyright @ 2011 Macmillan Publishers Ltd: [Nature Communication])

**Fig. 14:**
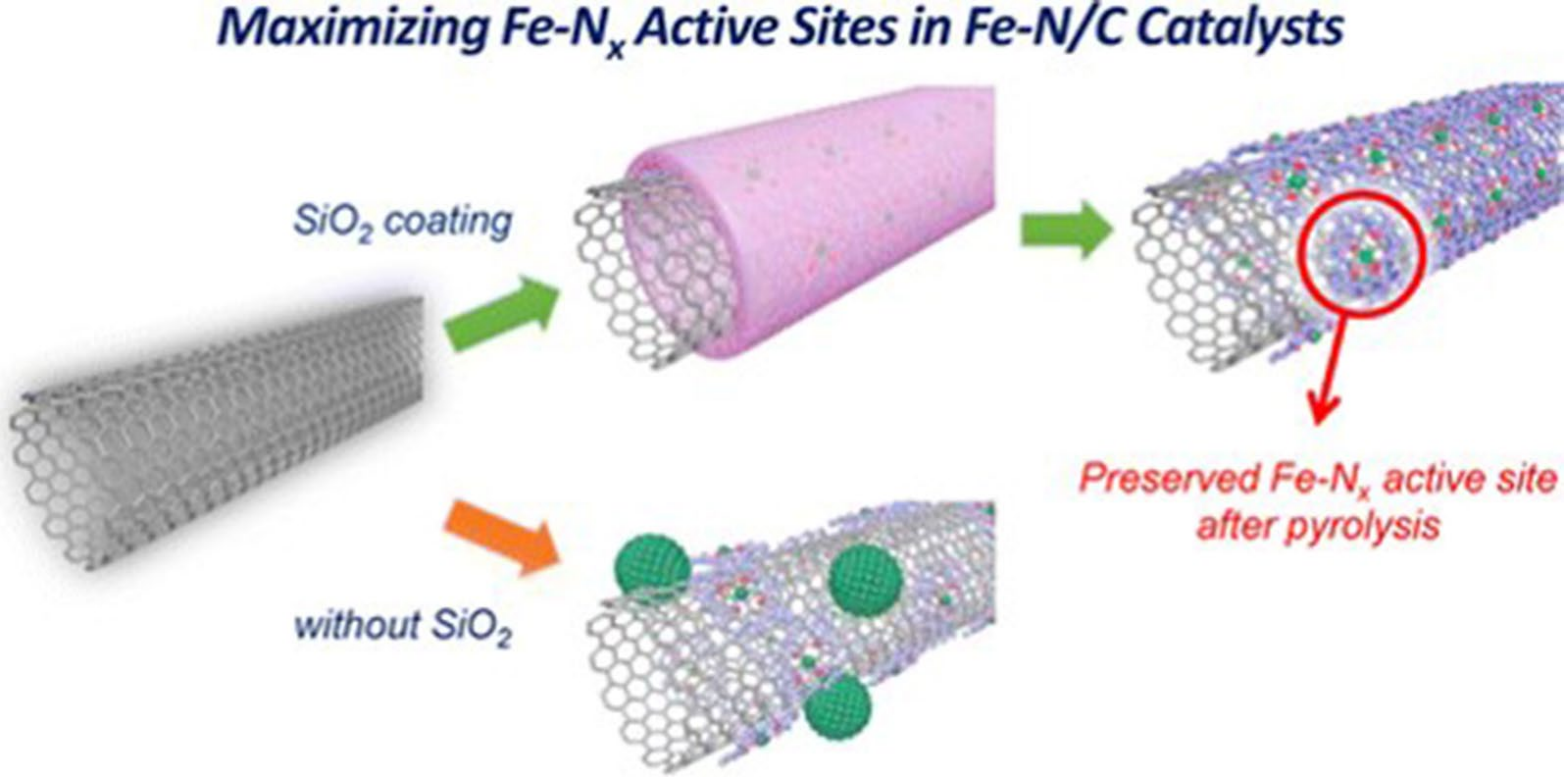
Fabrication process of Fe/PC/CNT with maximizing FeN_x_ active sites (reprinted with permission from Ref. [108] 2016 American Chemical Society)

Among various non-Pt based catalysts, the most promising material was transition metal/nitrogen supported with carbon (M/N/C, M=Co, Fe, Ni) catalysts, and they have been researched constantly after the Jasinski’s findings about co-phtalocyanine in 1964 [105]. The disadvantages of those materials in comparison with commercial Pt/C catalysts could be divided with three points of volumetric activity, mass transport ratio, and durability. The volumetric activities of M/N/C catalysts were merely 1/10 that of Pt–C catalysts in spite of recent progress [17, 106, 107], but this level could be sufficient to apply those materials to operate fuel cell, because the increase of input to electrode for retaining total activity was technically feasible. However, M/N/C structure has low mass transfer characteristics so that practical applications were restricted due to the insufficient power density. Proietti et al. achieved high power density using Fe-based catalysts [19], in which fabricated Fe/Phen/Z8 catalyst showed the power density comparable with the results of commercial Pt/C catalyst that loaded with 0.3 mg_pt_/cm^2^ as shown in Fig. 13. Also, improved long-term stability was achieved as only 15% degradation after operation test, though further researches required to provide sufficient performance for commercial applications. In 2016, novel results has been reported by Sa et al. [108]. As shown in Fig. 14, they proposed fabrication method based on silica-protective-layer for maximizing Fe–N_x_ active sites in catalysts. Iro prorphyrin precursor was adsorbed into CNT, then these complex were coated with silica layer. This coating was important step for preserving active sites during synthesis process. The coated layer was removed by etching, resulting in the formation of Fe–N/C catalysts which consisted of porphyrin carbon with FeN_x_ sites supported with CNT. Fabricated catalyst showed very high ORR activity and stability in alkaline media, and it achieved volumetric current density of 320 A/cm^3^ in acidic media that exceeds DOE target of 300 A/cm^3^ in 2020.

Encapsulated non-Pt metal based catalysts (ENPMC) have been considered as promising materials for fuel cell due to the advantages according to the protection of metal active sites with outer carbon layer [109–112]. The structure consisting of metal core and carbon shell by encapsulation process was typical concept for preserving active sites of metal. For example, Deng et al. reported Fe encapsulated pot-like CNT catalyst in 2013 [110], and fabricated materials showed low activities than M/N/C type catalysts, but remarkable enhancement of stability could be achieved. After operation test for 200 h, its activity was preserved similarly with initial value, and this result was caused by structural characteristics of encapsulated metal. Those kinds of materials have structural disadvantage related with access of reactant because active metal sites were not exposed in the catalyst surface, and further studies required to compliment low ORR activities. Recently, encapsulated Fe-based catalyst with high activity was proposed by Strickland et al. [99]. This complex was based on metal–organic-framework (MOF) as the template and FePhen organic precursor. This FePhen@MOF has well-ordered encapsulated structure with 2 nm thin film, and it showed high activity than commercial Pt/C catalyst in alkaline media and similar activity in acidic media.

Metal-free materials such as doped carbon were also important candidates to substitute commercial Pt-based catalysts. Basically, carbon-based materials did not have activity for ORR, but they have gained broad attentions from the result of Gong et al. in 2009 [113] that nitrogen doped CNT without metal exhibited high electrocatalytic activity and long-term stability. They first reported N-doped CNT as the promising candidates for metal-free electrocatalysts, and various research groups have been attempted to develop cost-effective metal-free catalysts based on similar structural concepts [114–116]. Recently, graphene has been noticed as promising materials to various applications resulted from high stability and surface area, and it has been applied to fabricate metal-free electrocatalysts [117, 118], in which novel materials with higher activities in comparison with N-doped CNT was reported that showed comparable activities with commercial Pt/C catalysts [119].

## Summary and future challenges

Development of novel electrocatalysts with low cost and high activity as an alternation of expensive Pt-based catalyst has been one of the most important topics in the field of PEMFC. In this review, we have highlighted recent findings based on the fabrication of nano-structured materials. Nanocomplexes composed of carbon-based materials and Pt showed high activity and long-term stability in comparison with commercial Pt/C catalysts, even though total amount of Pt loading was decreased. Also, Pt alloys exhibited synergetic effect that each disadvantage of Pt and non-Pt materials as a catalytic materials for fuel cell was counterbalanced by the formation of alloy. Non-Pt based catalysts have been considered as the most promising materials to replace conventional catalysts, recent findings showed possibility to practical commercial applications such as Fe–N/C, FePhen@MOF, and N-doped CNT. In spite of new findings above mentioned, carbon supported Pt nanoparticles are unique catalysts that can be utilized in fuel cell. Recent advances related with structural optimization of Pt-based catalyst showed potentials to solve cost problem, but there is obvious limitation in terms of market expansion. The desirable solution would be non-Pt based materials that have exhibited sufficient performance in recent results, but improvement of long-term stability is required for the commercial applications.
